# Application for Recognizing Sign Language Gestures Based on an Artificial Neural Network

**DOI:** 10.3390/s22249864

**Published:** 2022-12-15

**Authors:** Kamil Kozyra, Karolina Trzyniec, Ernest Popardowski, Maria Stachurska

**Affiliations:** 1 Ailleron SA, Jana Pawła II 43b, 31-864 Krakow, Poland; 2 Department of Machinery Exploitation, Ergonomics and Production Processes, University of Agriculture in Krakow, Balicka 116B, 30-149 Krakow, Poland; 3 Institute of Safety and Quality Engineering, Faculty of Management Engineering, Poznań University of Technology, J. Rychlewskiego 2, 60-965 Poznan, Poland

**Keywords:** convolutional neural networks, deep learning algorithm, machine image recognition systems

## Abstract

This paper presents the development and implementation of an application that recognizes American Sign Language signs with the use of deep learning algorithms based on convolutional neural network architectures. The project implementation includes the development of a training set, the preparation of a module that converts photos to a form readable by the artificial neural network, the selection of the appropriate neural network architecture and the development of the model. The neural network undergoes a learning process, and its results are verified accordingly. An internet application that allows recognition of sign language based on a sign from any photo taken by the user is implemented, and its results are analyzed. The network effectiveness ratio reaches 99% for the training set. Nevertheless, conclusions and recommendations are formulated to improve the operation of the application.

## 1. Introduction

Image recognition is a dynamically developing area for the use of artificial intelligence nowadays. Areas in which human work is successfully supported by modeling through the operation of artificial neural networks, including machine image recognition systems, include medicine [1,2,3], biology [4], agriculture [5,6,7,8,9,10,11,12], quality control [13,14], monitoring [15,16], astronomy [17,18] and many more. Convolutional neural networks are the tool used for these purposes [19,20]. Convolutional neural network architectures assume that the input is images. These networks consist of several convolutional layers and pooling layers that generalize and select the most necessary information for subsequent layers. The last element of the network is the block responsible for the flattening operation and application of the SoftMax activation function. The convolution layer performs an operation called “convolution”, which consists of multiplying a set of weights with the resulting input, similar to a traditional neural network. This layer is designed for two-dimensional input, and multiplication takes place between an array of input data and a two-dimensional array of weights, commonly known as a kernel or filter. Its task is to extract a specific value from a set, usually using the max-pooling or average-pooling functions [21,22]. This operation reduces the size of the output array. This is to extract the values that indicate the presence of the desired feature in the analyzed part of the image while removing the excess of unneeded information. The last block of the convolutional neural network architecture is a block whose task is to flatten the feature map to a one-dimensional array obtained after the pooling operation and to use the SoftMax activation function [23,24,25].

The aim of this research was the overall development and implementation of an application that recognizes sign language signs using a deep learning algorithm based on convolutional neural network architectures. For this purpose, a module that converts photos to a numerical form was prepared. Subsequently, the appropriate network architecture was selected, and a model was developed, which was subjected to the learning and testing process. The effectiveness of machine recognition of a single image was verified using a set of photos with high variability in terms of background types, lighting and sharpness. There are many examples in the literature of the use of visual gesture recognition techniques for control. Some examples of the use of hand gesture recognition for control include: a wheelchair [26], the flight of unmanned aerial vehicles [27], the work of an industrial robot [28], the work of a robot servo drive and intelligent home devices [29]. In the e-science database, there are at least several dozen studies in which the problem of recognizing gestures in sign language using neural networks has been solved. This method has been implemented to recognize Arabic, Hindu, Bangla and other characters. In many cases, the CNN classifier is used, as we have done in this study. Shanta et al. [30] tried to implement a Bangla sign language system that uses SIFT function extraction and a convolutional neural network (CNN) for classification. They also showed that using the SIFT function increases the accuracy of CNN in detecting Bangla sign language. The Al Rashid Agha [31] team published an article that provides a comprehensive study of the various approaches and techniques used to advance the science of sign language recognition. The systems that were developed used a support vector machine (SVM), a K-nearest neighbours (KNN) classifier, deep convolutional neural networks (CNNs) and artificial neural networks (ANNs). Mustafa [32] published a similar study. It was aimed at reviewing sign language recognition systems based on various classifier techniques. Mainly, neural networks and deep learning classifiers have been used to identify different sign languages, and this survey aimed to review the best classifier model representing sign language recognition (SLR). The author implemented numerous classifiers into the SLR system, such as CNN, RNN, MLP, LDA, HMM, ANN, SVM, KNN and others. Each classifier was validated for the image recognition accuracy to determine which deep learning-based classifiers were the best at recognizing sign language signs.

## 2. Materials and Methods

**Figure 1:**
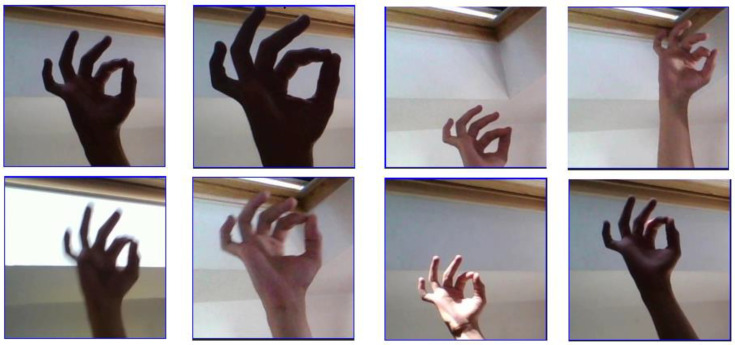
Test set visualization diversity for the letter F.

Python version 3.7.2, PyCharm development environment and the Keras and TensorFlow libraries for creating, training and presenting the test results of the convolutional neural network were used to create the model. Additionally, the Flask framework was used to create the web application. The training set consisted of 50,000 photos with dimensions of 200 × 200 pixels, consisting of 26 characters of American Sign Language letters and downloaded from the online data science platform Kaggle. In the photos, the hand presenting the sign language gesture (interpreted in sign language as a sign of the alphabet) is presented in different lighting and in a different configurations. Examples of photos for a single letter are shown in Figure 1. 

Operation of the data loading module is based on the load_data method that takes two arguments: size—the number of images from the test set that will be analysed in the process of training and testing the network, and test_split—the ratio of the size of the training set to that of the test set. The following values were adopted for these variables: 50,000 and 0.2, respectively. Further, the initial dataset was systematized. As a result, a collection of photos sorted by each character was created. Prior to data input into the neural network, the ordered set elements were mixed using the random.shuffle function (). To significantly shorten the calculation times and while preserving important data stored in the images, the size of each photo was reduced from 200 × 200 pixels to 50 × 50 pixels using img.resize (). The set created in this way was then divided into two subsets: training and test, where the x_train_images and x_test_images arrays contained images, and y_train_labels and y_test_labels were the corresponding labels. A value in the range <0, 26> was assigned to each element of the y_train labels, corresponding to the letters A, B, C, …, Z. As Keras framework models do not accept labels in this format, we used a hot encoding procedure to convert each of the values of the letter category (A, B, C … Z) into a new column and assigned a value of 0 or 1 to it.

## 3. Implementation of the Application

### 3.1. Development of an Artificial Neural Network Model

The architecture of the implemented artificial neural network includes two convolutional layers, followed by layers responsible for pooling. The dense layer is placed at the very bottom. 

The parameters of the method that creates the convolution layer include the number of filters and their sizes and the type of activation function. Moreover, the first layer takes an argument specifying the shape of the input array. The multitude of filters allows the extraction of more elements characteristic of a given image, a consequence of which the network achieves better results when classifying images. To solve the described problem, 32 filters in the first convolution layer and 64 filters in the second were used. Filter size was assumed to be 5 × 5 pixels. At the same time, the ReLU function was selected as the activation function. The input_shape variable had the value (50, 50, 3) because the images used were 50 × 50 px and they were coloured, and the RGB model has three channels. It is also important that the value of this parameter should not be too large in relation to the size of the processed image because increasing the size of the filter reduces the resolution. Hence, more information relevant to subsequent layers is lost; the flattening operation was necessary to perform. This action was necessary because the dense layer, which is the last element of the convolutional neural network, requires a one-dimensional array. The last of the network layers (dense) returns an N-dimensional array, where N is the number of classes among which we recognize the images. In the case of this project, it is the 26 different letters of the alphabet. For each class, the network outputs the probability of belonging to that class of a given character. For example: assuming that characters from the set [A, B, C] are recognized and the letter B is recognized with a probability of 90%, the output network returns the array [0.03, 0.9, 0.07]. 

### 3.2. Compilation, Training and Tests of the Neural Model

Compilation of the created convolutional neural network consisted of calling the built-in compile () method, in which the rmsprop optimizer and the so-called loss function, categorical_crossentropy, were specified. This is widely used to solve image classification problems [31,32].

Training of the created neural network model was carried out by calling the fit () method. This function accepts four parameters, namely a set of training images, a set of labels, the percentage size of the data set to be validated after each training iteration (called validation_split) and the number of training iterations (called epochs). The value of the last parameter was set to 18 by default. 

After compiling and training, the artificial neural network model should correctly recognize the signs of the sign language alphabet from the photos. A built-in evaluate () method was used to verify the correct operation of the network. The arguments of the function are a set of images and labels, and the result of its launch is the percentage value of the test result of 0.9845. The final step was to save the trained model to a file using save (), which made it accessible for use in an application that recognizes any image.

### 3.3. Demonstration Module for Recognizing Any Photo

**Figure 2:**
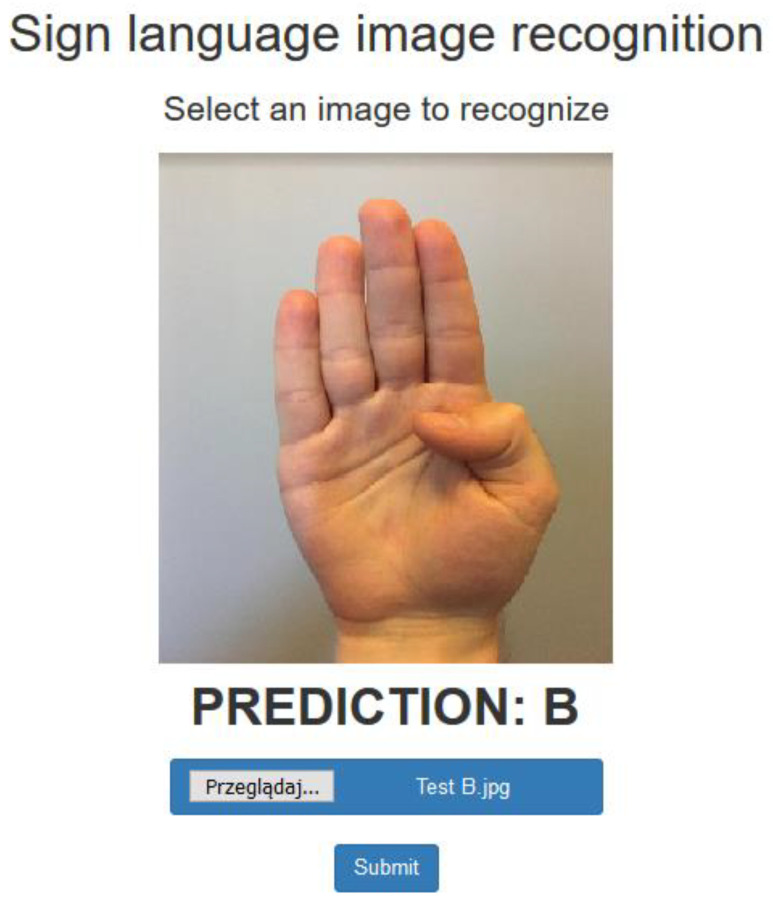
Visualization of the application interface showing the verification result.

Export of the trained model to a file with the .h5 extension made it possible to create an application that allows it to recognize gestures of the sign language alphabet. The created application is a web-based and consists of three states. The first is the so-called “empty state” and is presented to the user upon entering the website if no image has been selected before. The second one becomes visible after clicking the “Browse” button and selecting the photo that should be recognized. The application accepts photos of any size with square proportions (before the photo is uploaded to the neural network, it is automatically converted to 50 × 50 pixels) and the *.jpg extension. The possibility of implementing any photo into the application allows users to verify its operation on real and more diverse data. After selecting the file and clicking the “Submit” button, the photo is processed by the artificial neural network, and the processing result is presented in the third state. Below is a photo entered by a user and the network’s answer as a predicted letter of the alphabet (Figure 2).

## 4. Results

### 4.1. Visualization and Verification of University Neural Network Results

**Figure 3:**
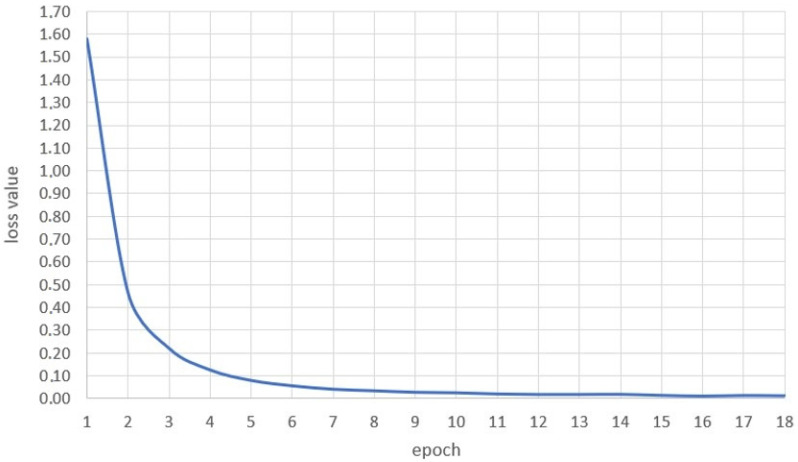
Loss function curve of the network learning process.

The primary parameter verifying the usefulness of the network is the so-called loss-function curve, shown in Figure 3. It returns information about the results of the network learning process. If the network prediction results in misses, the value of the loss function is high; otherwise, it is low. The estimated function value is expected to decrease with each successive learning cycle. The graph presented in the figure represents that the loss function for the analysed network in the first learning cycle has a high value that decreases with each run cycle and approaches zero. The desired result was achieved when the number of missed responses of the neural network decreased with each learning cycle.

**Figure 4:**
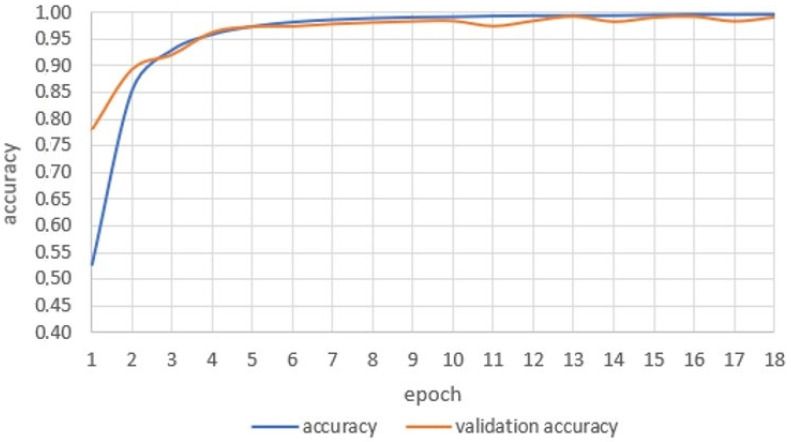
Comparison of the validity function of the answer with the validity function of the validation set.

The parameter that allows determination of the ability of the neural network to assimilate knowledge in the fastest and easiest way is the accuracy of the forecasts made. This is the quotient of the number of correctly recognized datapoints out of the total number of trials. Figure 4 shows a comparison of the validity function values with the values of the validation set.

Starting from the third learning cycle, the values of both functions are similar. Based on the obtained results, it may be concluded that there is no overfitting phenomenon in the analysed network, and thus the network should have a high ability to recognize unknown objects. The created neural network was validated after the learning process. The size of the test set was specified in the input module: the network uses 50,000 photos, and the value of the test set was 20%, i.e., 10,000 photos. With the presented architecture and the assumed parameters of the neural network, the accuracy of image recognition from the training set reached 99% correctness.

### 4.2. Identifying Unknown Images

**Figure 5:**
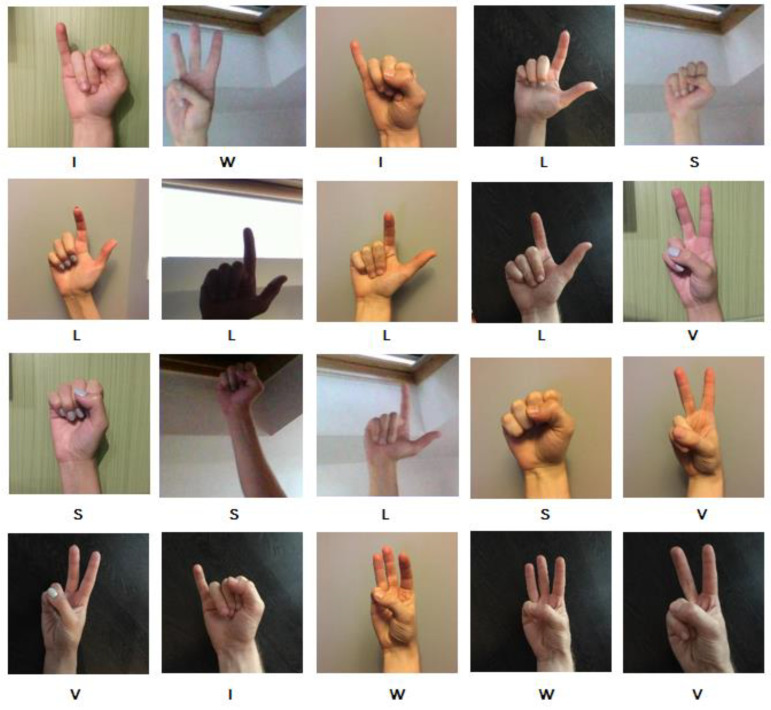
Part of the set of photos used for testing the application.

The suitability of the network for recognizing characters from previously unknown images was checked. For the purpose of this activity, 40 photos with information about the characters “L”, “I”, “W”, “V” and “S” were entered as the network input. The network was tested using an application developed for this work. Similar to the previous exercise, the photos of each letter show the hand with different backgrounds and under different lighting. A sample of the test data is shown in Figure 5.

**Figure 6:**
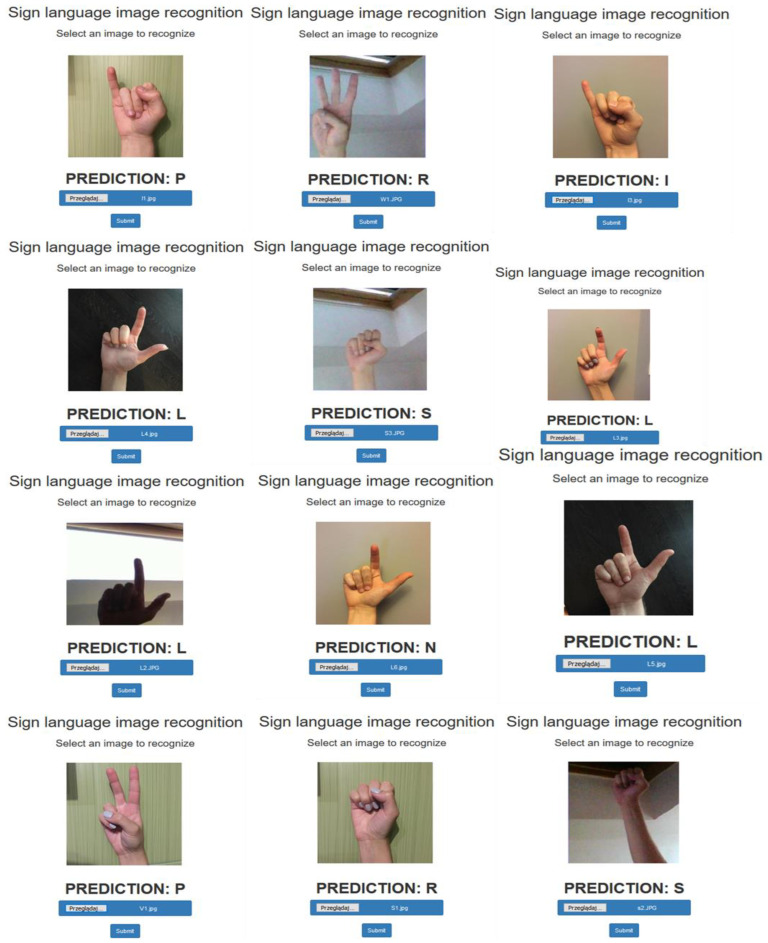
A sample of recognition results of images unknown to the network.

The image recognition results presented in Figure 6 were obtained for the selected photos.

**Figure 7:**
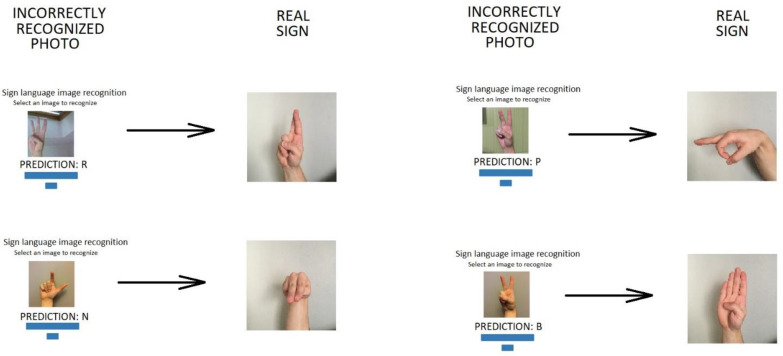
Examples of incorrectly recognized sign language gestures.

The presented photos show that 24 out of 40 analysed signs were correctly recognized. Figure 7 shows incorrectly recognized photos and their comparison with the real sign.

The test set includes photos taken on a green striped background. None of these were properly recognized. In terms of numbers 3 and 8, the character “P” was recognized, and for the number 4, the character “R”. An error for number 4 may have been the result of the similarity between the real “S” sign and that recognized by the neural network as “R”. For both signs, the thumb is placed the same way, although the letter “S” has a fully closed fist whereas for the letter “R” it is partially open. A similar situation occurs with misdiagnoses of 1, 6 and 7. Likewise, in these cases, there are similarities between the input character and the recognized character: each input character “W”, “V” and “I” requires showing one, two or three fingers, while the remainder stay joined. It is worth noting that, nevertheless, hand gestures for letters “R”, “B” and “J” remain similar to those for “W”, “V” and “I”, yet these were correctly identified. 

## 5. Conclusions

This paper focused on the development of a convolutional artificial neural network model capable of recognizing sign language gestures based on a static picture. The artificial neural network model was created from two convolutional layers responsible for pooling plus a dense layer. For training and testing purposes, a set of 50,000 photos was used, where 40,000 of these were taken as training material and 10,000 were used for checking the learning results. The effectiveness of the training set reached 99%. The Python language and the Keras library were used to create the neural network model and for training and testing, while the PyCharm was the programming environment. Based on the network operation, a web application was also prepared to test the network’s skills. It allows successful recognition of gestures of the sign language alphabet from a photograph taken by any user. The application was tested for actual use, i.e., recognition of photos not belonging to the training set. As a result of this examination, 24 out of 40 images were identified. It is worth noticing that a large number of misidentified signs are photographs taken against an atypical background, for example, vertical stripes with a width similar to the width of fingers making gestures indicating a specific sign of sign language. The sharpness of the photos and the lighting used are certainly also responsible for reducing the effectiveness of character recognition. However, the authors deliberately used such a testing set because, ultimately, the application will be used in conditions where it is difficult to ensure appropriate image quality. In some cases, the artificial neural network partially recognized the image, which means that in the recognized photo and the actual letter, the position of the hands was similar. For example, the character representing the letter V is similar to the character representing the letter W. The only difference is the addition of one finger. The dataset used in this project contained photographs of one hand making the gestures. Greater variation in test data would certainly have an impact on the test results. Nevertheless, it should be noted that the photos did not undergo any additional processing apart from resizing. Bear in mind that the use of a uniform background in the photos entered into the application or the implementation of a module responsible for removing as much irrelevant information as possible from the photo before delivering it to the artificial neural network may improve its accuracy [33,34]. 

The authors intend to implement the developed application for a vision system installed in a tractor cabin. The selected gestures will then be used to perform certain procedures, such as, for example, raising the aggregated machine working in the soil or changing its operating parameters.

## Data Availability

The data presented in this study are available upon request from the respective author. The data are not publicly available due to the possibility of their commercial use by the unit in which the authors are employed and for the use of the source data for further research and development by an accredited laboratory (Polish accreditation number AB 1698) whose staff are the authors of this publication.
